# Supplementary figures and images for: Isolation and identification of a halophilic and alkaliphilic microalgal strain
Source: PeerJ. 2019 Jun 24;7:e7189. doi: 10.7717/peerj.7189 (PMC6596407; doi:10.7717/peerj.7189)

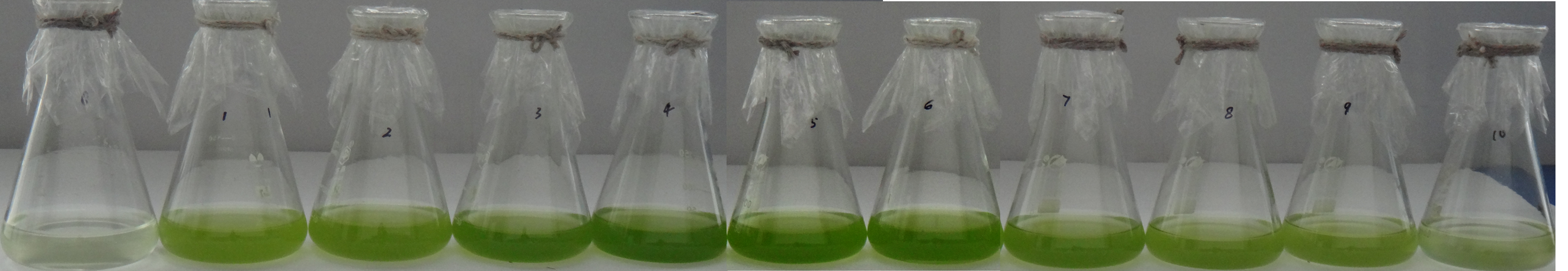

Supplement: Supplemental Information 1 — Microalgal growth under different NaHCO3 concentrations. The final concentrations of NaHCO3 were 0, 100, 200, 300, 400, 500, 600, 700, 800, 900 and 1000 mmol/L. The green color of the algal cells darkened as the NaHCO3 concentration in the medium increased, indicating that cell growth significantly accelerated. When the NaHCO3 concentration was 400 mmol/L, the growth rate was the greatest. [file peerj-07-7189-s001.png]

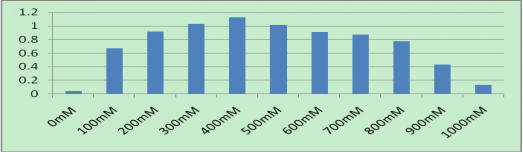

Supplement: Supplemental Information 2 — The final concentrations of NaHCO3 were 0, 100, 200, 300, 400, 500, 600, 700, 800, 900 and 1000 mmol/L. When the NaHCO3 concentration was 400 mmol/L, the growth rate was the greatest. So was the value of ODλ=700 detected by a UV spectrophotometer, indicating the peak of the exponential growth phase. [file peerj-07-7189-s002.png]

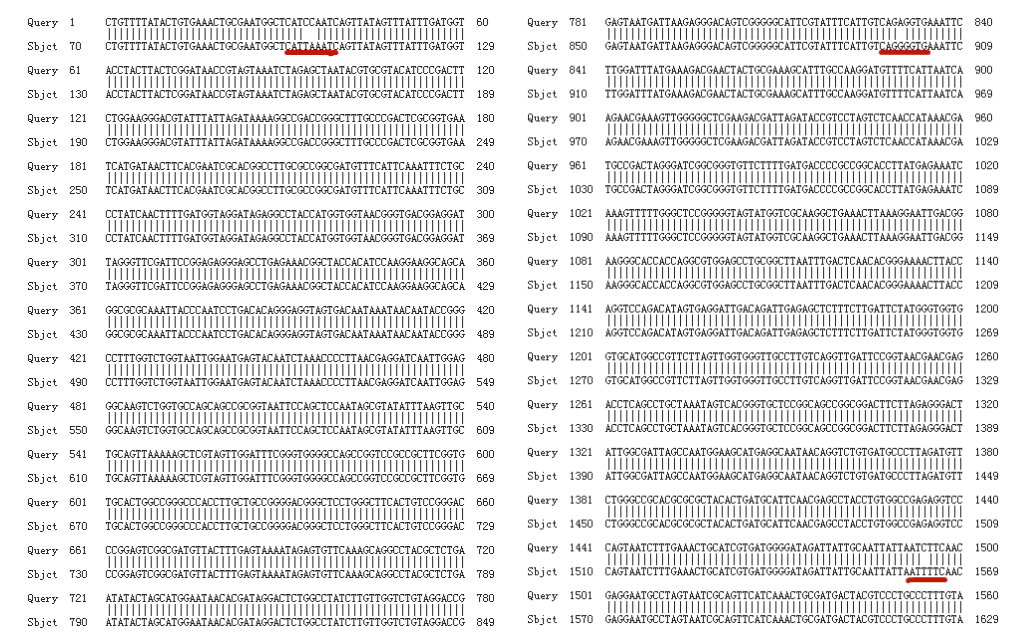

Supplement: Supplemental Information 3 — The nucleotide sequence was submitted to GenBank. Homologous comparison with the data of GenBank showed that it has 99% similarity to Nannochloris sp. BLD-15, with only four base substitutions. [file peerj-07-7189-s003.png]

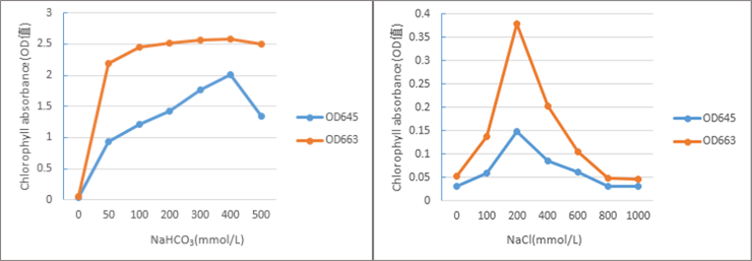

Supplement: Supplemental Information 4 — Chlorophyll absorbance under different NaHCO3 and NaCl concentrations. When the NaHCO3 concentration was 400 mmol/L, the chlorophyll absorbance was the greatest. When the NaCl concentration was 200 mmol/L, the chlorophyll absorbance was the greatest. [file peerj-07-7189-s004.png]

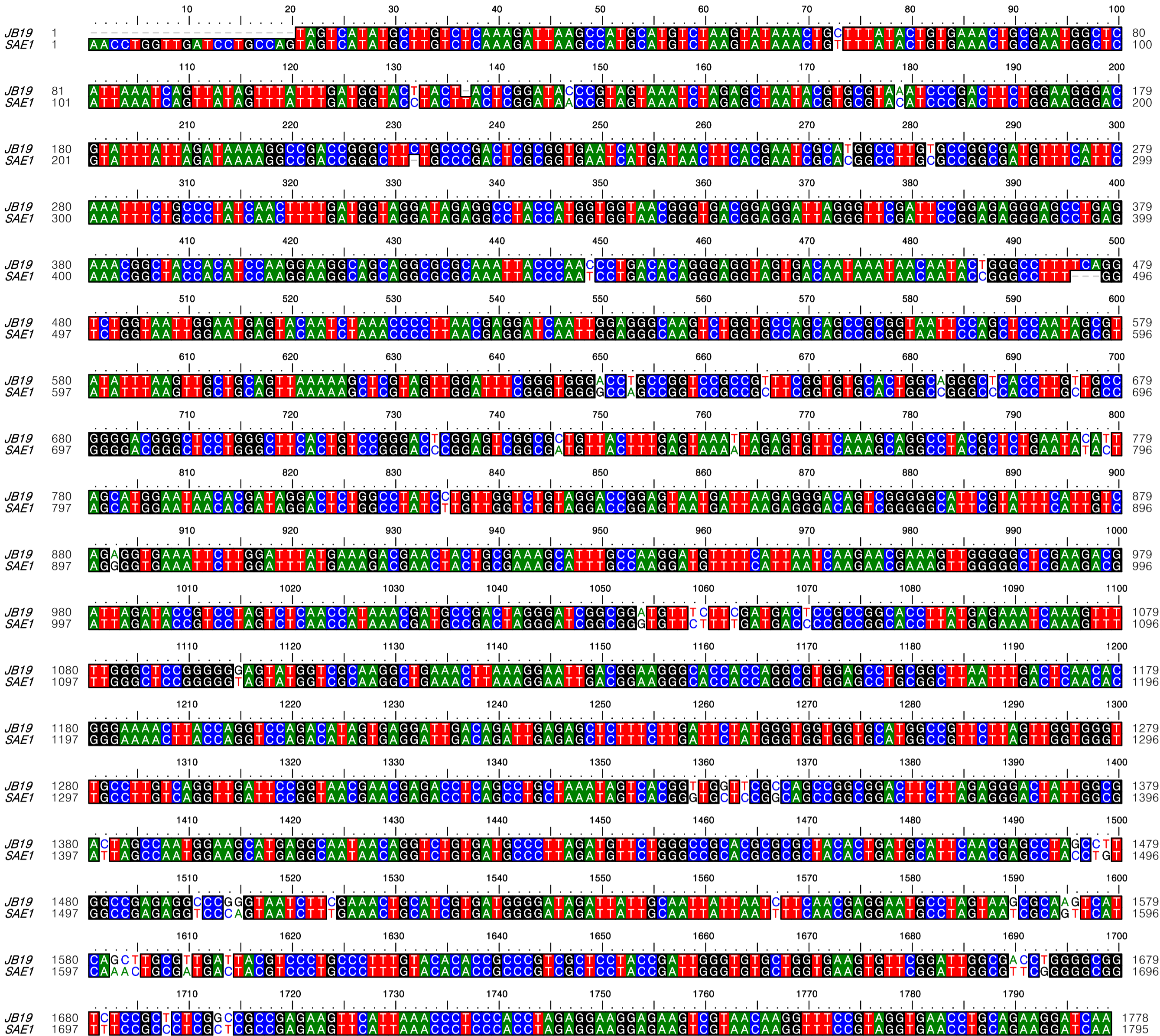

Supplement: Supplemental Information 7 [file peerj-07-7189-s007.pdf]
